# Acquired Hemophilia A and ST-Elevation Myocardial Infarction (STEMI): A Rare Presentation and Management Dilemma

**DOI:** 10.7759/cureus.38549

**Published:** 2023-05-04

**Authors:** Jevin Yabut, Gian Lima, Ritika Vankina, Swarup Kumar, Joyce Meng

**Affiliations:** 1 Medicine, University of Connecticut, Farmington, USA; 2 Internal Medicine, University of Connecticut, Farmington, USA; 3 Hematology and Oncology, University of Connecticut, Farmington, USA; 4 Cardiology, University of Connecticut, Farmington, USA

**Keywords:** factor vii replacement, factor vii, novoseven, acute coronary syndrome, factor viii deficiency, coagulopathy, stemi, hemophilia a

## Abstract

Thrombotic events are a rare complication of recombinant activated factor VII (rFVIIa) therapy in patients with hemophilia. We present a case of a 71-year-old male who developed ST-elevation myocardial infarction after receiving rFVIIa replacement therapy for acquired hemophilia A.

## Introduction

This article was previously presented as a poster at the 2022 American Heart Association Conference on November 7, 2022.

Acquired hemophilia A (AHA) is a rare autoimmune bleeding disorder in which antibodies attack clotting factor VIII. Recombinant activated factor VII (rFVIIa) is an agent that bypasses the acquired factor VIII deficiency and has been approved by the FDA for the treatment of bleeding and perioperative management in AHA. Thrombotic events are a rare complication of the use of this therapy [1]. We present a case of ST-elevation myocardial infarction (STEMI) after receiving rVIIa replacement therapy for AHA.

## Case presentation

**Figure 1:**
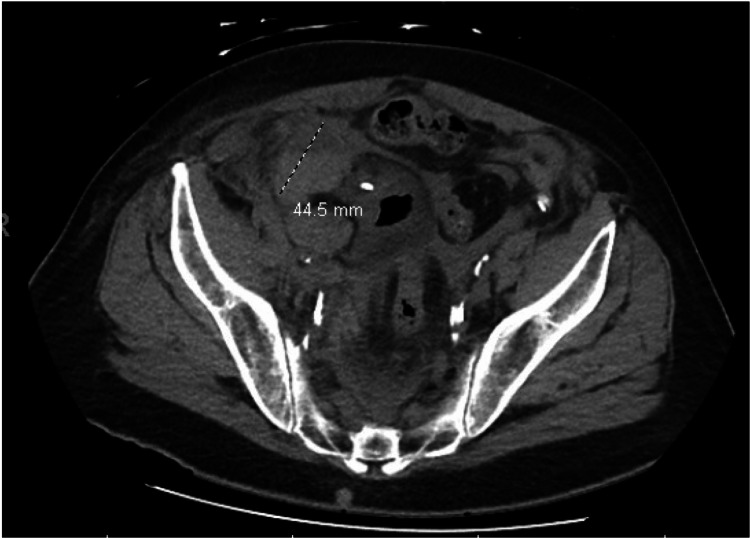
CT abdomen and pelvis demonstrating hemoperitoneum and multilocular hematoma to the right of ureteral implantation

A 71-year-old male with a past medical history of hypertension, diabetes, hyperlipidemia, and low-grade, non-invasive right ureteral urothelial carcinoma presented for elective robotic right distal ureterectomy, psoas hitch with ureteral reimplantation, and pelvic lymph node dissection. The patient was noted to have persistent hematuria draining from his Foley catheter. CT of the abdomen revealed hemoperitoneum and multiple hematomas next to the ureteral implant (Figure 1).

**Figure 2:**
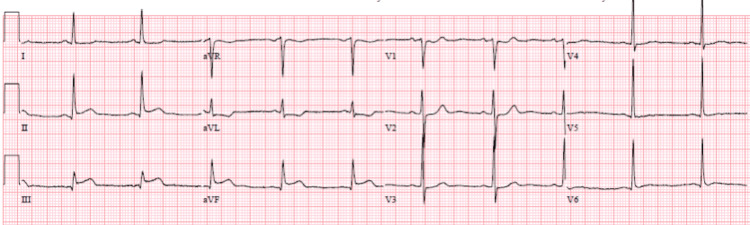
ECG demonstrating ST elevations in leads II, III, and aVF

He remained stable hemodynamically but required multiple blood transfusions. Subsequent evaluation for coagulopathy found evidence of acquired factor VIII deficiency. The patient received FVIII replacement therapy but failed to respond due to high levels of factor VIII inhibitors. His coagulopathy improved after being placed on rFVIIa therapy. After 20 days of therapy, he was noted to have ST changes on telemetry. He was asymptomatic. The patient’s blood pressure was 139/63 mmHg, heart rate 72 bpm, and he was saturating well on room air. The physical exam was unremarkable. ECG showed ST elevations in leads II, III, and aVF (Figure 2).

**Figure 3:**
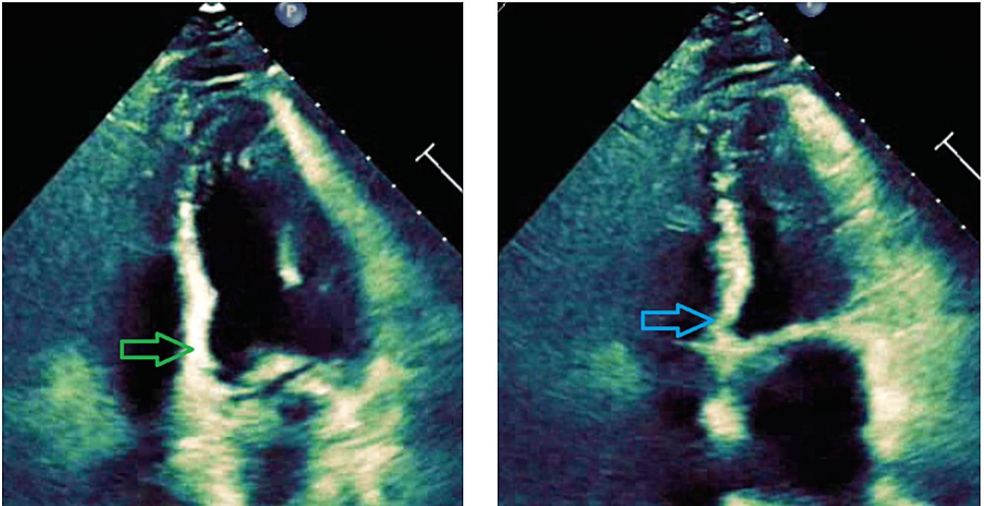
Transthoracic echocardiogram apical 4 -chamber view showing akinesia of the basal inferoseptal wall. Abnormal motion during diastole (green arrow) and systole (blue arrow)

Transthoracic echocardiogram showed a left ventricular ejection fraction of 55% with nearly akinesis of the basal inferoseptal wall (Figure 3).

**Figure 4:**
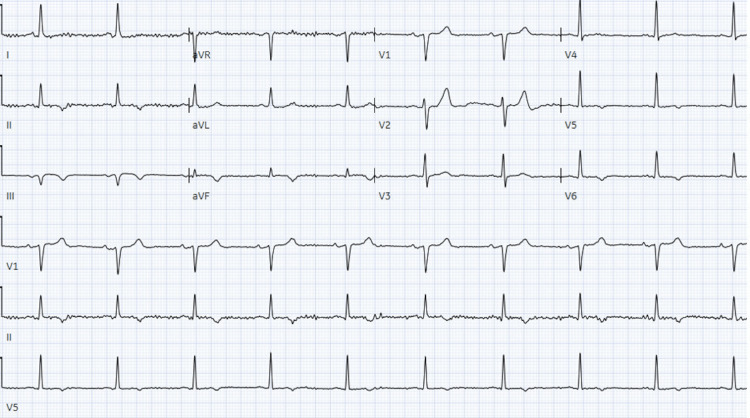
ECG demonstrating serial evolution of Inferior myocardial infarction with T wave inversions in leads II, III, and aVF

Serial ECGs demonstrated an evolution of an inferior myocardial infarction. Maximal troponin I was 48.86 ng/mL (Figure 4).

rFVIIa was held due to its thrombotic risk. After a multidisciplinary discussion, the decision was made not to start anticoagulation nor perform coronary angiography given that the patient was asymptomatic, hemodynamically stable, and had severe coagulopathy. He was started on aspirin without obvious bleeding complications.

The patient remained asymptomatic from a cardiac standpoint. He had no arrhythmias on telemetry. He did not have ongoing bleeding complications. He was discharged with plans to follow up in the cardiology clinic.

## Discussion

AHA is a rare autoimmune bleeding disorder in individuals with no history of bleeding disorders in which antibodies (known as inhibitors) attack clotting factor VIII. In the United States, AHA is estimated to affect 0.2-1 individuals per 1,000,000/year and is predominantly a disease of the elderly [2]. It is associated with underlying autoimmune conditions and malignancy; however, 50% of cases are idiopathic [3]. Bleeding events can range from minor (hemarthrosis, epistaxis) to severe (intracranial bleeding). Individuals with an isolated prolonged activated partial thromboplastin time, normal prothrombin time, decreased factor VIII levels, and increased titer count of factor VIII inhibitors are diagnosed with the disease.

rFVIIa is an agent that bypasses the acquired factor VIII deficiency. The FDA has approved it for the treatment of bleeding and perioperative management in AHA. rFVIIa induces hemostasis by enhancing thrombin generation on the thrombin-activated platelet surface to generate a fibrin hemostatic plug [4]. Thrombotic events are a rare complication of therapy. In a retrospective study of 12,288 bleeding and surgical episodes, the overall rate of thrombosis was 0.17%. The most common risk factors were age ≥65 years (29%) and concomitant cardiac or vascular disease (18%) [1].

The prognosis for patients with hemophilia (PWH) has improved dramatically with the introduction of clotting factor concentrates; their median survival is now only slightly less than the general population. As a result, PWH has a higher prevalence of concomitant illnesses such as coronary artery disease. PWH tends to have fewer ischemic complications compared to their counterpart with similar cardiac conditions; this can be attributed to their hypercoagulable state and reduced thrombus formation [3].

Treatment of acute coronary syndrome (ACS) in patients with PWH is challenging due to the increased risk of bleeding after percutaneous coronary intervention (PCI). There is currently no universally accepted protocol for management. A multidisciplinary discussion between cardiologists and hematologists is necessary to tailor the treatment approaches to each individual patient. In relatively low-risk ACS patients, a conservative approach can be considered [5]. Patients with moderate- to high-risk ACS need early revascularization. Depending on the severity of the bleeding, patients may need concurrent clotting factor replacement therapy supervised by a hemophilia specialist.

Anticoagulation can be considered after factor replacement therapy depending on the thrombotic risk of the patient [3]. Anticoagulation without factor replacement therapy is not recommended [1]. Unfractionated heparin is preferred due to its short half-life and reversibility by protamine administration [6]. Hemophilia is not associated with platelet abnormalities; hence, antiplatelet agents can be administered. Dual antiplatelet therapy is recommended post-PCI to prevent stent thrombosis.

## Conclusions

AHA is an autoimmune bleeding disorder in which antibodies are produced that target clotting factor VII. Thrombotic events, such as ACS, should be considered in patients undergoing rFVIIa therapy. Treatment of ACS in PWH is challenging given the increased bleeding risk. A multidisciplinary approach involving hematologists and cardiologists should be taken when managing PWH who present with ACS.
